# A statistical framework for evaluating the repeatability and reproducibility of large language models

**DOI:** 10.1101/2025.08.06.25333170

**Published:** 2025-11-04

**Authors:** Cathy Shyr, Boyu Ren, Chih-Yuan Hsu, Rory J. Tinker, Thomas A. Cassini, Rizwan Hamid, Adam Wright, Lisa Bastarache, Josh F. Peterson, Bradley A. Malin, Hua Xu

**Affiliations:** 1 Department of Biomedical Informatics, Vanderbilt University Medical Center, 2525 West End Avenue, Nashville, 37203, TN, USA.; 2 Department of Biostatistics, Vanderbilt University Medical Center, 2525 West End Avenue, Nashville, 37203, TN, USA.; 3 Department of Pediatrics, Vanderbilt University Medical Center, 2200 Children’s Way, Nashville, 37232, TN, USA.; 4 Laboratory for Psychiatric Biostatistics, McLean Hospital, 115 Mill Street, Belmont, 02478, MA, USA.; 5 Department of Medical Genetics and Genomics, Icahn School of Medicine at Mount Sinai, 1 Gustave L. Levy Pl, New York, 10028, New York, USA.; 6 Department of Medicine, Vanderbilt University Medical Center, 1161 21st Ave S, Nashville, 37232, TN, USA.; 7 Department of Computer Science, Vanderbilt University, 1400 18th Avenue S, Nashville, 37212, TN, USA.; 8 Department of Biomedical Informatics and Data Science, Yale School of Medicine, 101 College Street, New Haven, 06520, CT, USA.

**Keywords:** repeatability, reproducibility, large language model, artificial intelligence, diagnostic reasoning

## Abstract

A major concern in applying large language models (LLMs) to medicine is their reliability. Because LLMs generate text by sampling the next token (or word) from a probability distribution, the stochastic nature of this process can lead to different outputs even when the input prompt, model architecture, and parameters remain the same. Variation in model output has important implications for reliability in medical applications, yet it remains underexplored and lacks standardized metrics. To address this gap, we propose a statistical framework that systematically quantifies LLM variability using two metrics: repeatability, the consistency of LLM responses across repeated runs under identical conditions, and reproducibility, the consistency across runs under different conditions. Within these metrics, we evaluate two complementary dimensions: semantic consistency, which measures the similarity in meaning across responses, and internal stability, which measures the stability of the model’s underlying token-generating process. We applied this framework to medical reasoning as a use case, evaluating LLM repeatability and reproducibility on standardized United States Medical Licensing Examination (USMLE) questions and real-world rare disease cases from the Undiagnosed Diseases Network (UDN) using validated medical reasoning prompts. LLM responses were less variable for UDN cases than for USMLE questions, suggesting that the complexity and ambiguity of real-world patient presentations may constrain the model’s output space and yield more stable reasoning. Repeatability and reproducibility did not correlate with diagnostic accuracy, underscoring that an LLM producing a correct answer is not equivalent to producing it consistently. By providing a systematic approach to quantifying LLM repeatability and reproducibility, our framework supports more reliable use of LLMs in medicine and biomedical research.

## 1 Introduction

Large language models (LLMs) are artificial intelligence (AI) systems capable of learning complex language patterns and generating human-like text. They have demonstrated promising performance across a range of clinical applications, including drafting hospital discharge summaries [1], responding to patient messages [2], and providing diagnostic support [3]. Among these, diagnostic support has gained increasing attention, with recent studies demonstrating strong LLM performance in diagnostic reasoning and clinical decision-making [4–9]. However, prior studies focused primarily on diagnostic accuracy as the key performance metric. Other properties essential for the responsible use of AI in medicine, such as the repeatability and reproducibility of LLM responses, remain underexplored.

Repeatability is defined as the agreement of model outputs under identical conditions, and reproducibility the agreement under pre-specified, different conditions (e.g., different users or experimental setup) [10]. Because LLMs generate text by sampling the next token (or word) from a probability distribution, the stochastic nature of this process can lead to different outputs even with the same input prompt, model, and parameters. Variability is common to human reasoning in medicine, as clinicians may reasonably arrive at different conclusions depending on their specialty; the key distinction is that clinicians can explain their reasoning and contextualize variability [11], whereas LLMs generate outputs without a rigorous way to characterize their variability. This can raise concerns in diagnostic settings, as an LLM can generate divergent diagnostic suggestions for the same patient case across multiple runs, potentially undermining clinician trust and limiting the model’s utility for decision support [12, 13]. Such concerns reflect the broader challenge in AI evaluation, and the U.S. Food and Drug Administration (FDA)’s draft guidance on AI-enabled medical software recommends quantifying model variability based on repeatability and reproducibility [10]. These principles align with broader calls by medical journals to assess variability as a core component of AI evaluation in medicine and biomedical research [14].

In this study, we present a general statistical framework for evaluating LLMs’ repeatability and reproducibility. Our framework provides a systematic way to assess these two properties across two dimensions, semantic and internal, yielding four complementary metrics: 1) Semantic Repeatability, 2) Internal Repeatability, 3) Semantic Reproducibility, and 4) Internal Reproducibility. Semantic metrics measure the variability in the *meaning* of outputs across repeated runs. By contrast, internal metrics measure *variability in the model’s token*-*level probability distributions*, which reflects the underlying stability of the model’s word-generation process. These measures are particularly important in clinical settings, where small shifts in meaning or phrasing may influence clinician interpretation and downstream decision-making [15]. In contrast to studies that focused on assessing the *accuracy* of LLMs’ responses (e.g., diagnostic accuracy [3, 6] or hallucination detection [16–18]), our approach systematically quantifies response *variability* to provide a complementary assessment of LLM reliability.

We applied this framework to different LLMs (both open source and commercial), diagnostic reasoning prompts, and two datasets representing distinct clinical problem spaces: 1) standardized benchmark questions from the U.S. Medical Licensing Examination (USMLE) [19], and 2) real-world rare disease patient cases from the National Institutes of Health (NIH) Undiagnosed Diseases Network (UDN) [20]. USMLE questions reflect common, prototypical clinical scenarios with clear diagnostic pathways and a single best answer, while UDN cases have rare, diagnostically challenging presentations characterized by incomplete information, atypical findings, and greater diagnostic uncertainty. By spanning both prototypical and real-world patient cases, our evaluation assesses LLM repeatability and reproducibility across a broad spectrum of clinical presentations to provide insights into LLMs’ reliability in both routine and diagnostically challenging scenarios in medicine.

The proposed framework can support multiple facets of clinical AI tool development and oversight, including model selection, prompt design, and readiness assessment for clinical integration. Because the proposed metrics are agnostic to both the LLM and input prompt, the framework is broadly generalizable to other use cases beyond diagnostic reasoning. By quantifying the repeatability and reproducibility of LLMs’ responses, our work enables a more comprehensive assessment of model performance and supports efforts toward the development of reliable AI applications in medicine and biomedical research.

## 2 Methods

### 2.1 Definitions of Repeatability and Reproducibility

**Fig. 1:**
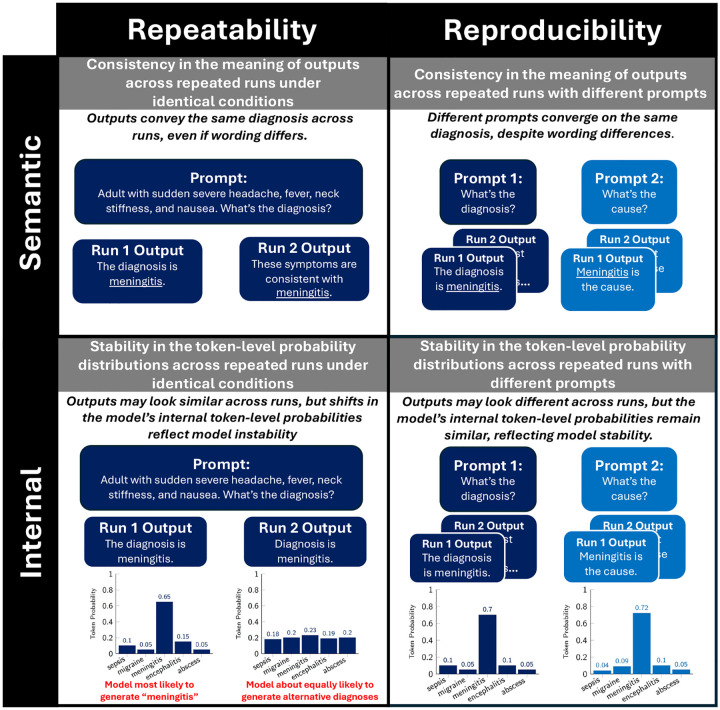
Overview of metrics. (1) Semantic Repeatability: consistency of meaning across repeated runs under identical conditions. (2) Semantic Reproducibility: consistency of meaning across repeated runs with different prompts. (3) Internal Repeatability: stability of token-level probability distributions across repeated runs under identical conditions. (4) Internal Reproducibility: stability of token-level probability distributions across repeated runs with different prompts.

Motivated by the FDA’s draft guidance on AI-enabled medical software, which recommends evaluation of both repeatability and reproducibility in AI systems [10], our framework defines and operationalizes four metrics: 1) Semantic Repeatability, 2) Internal Repeatability, 3) Semantic Reproducibility, and 4) Internal Reproducibility (Figure 1). Repeatability is defined by the FDA as “*the closeness of agreement of repeated measurements taken under the same conditions*” [10]. In our framework, repeatability corresponds to generating LLM responses across repeated runs using the same model, prompt, and generation parameters (e.g., temperature and top-k) for the same clinical case, and measuring the agreement across these responses. In contrast, reproducibility is defined as “*the closeness of agreement of repeated measurements taken under different, pre*-*specified conditions*” [10]. In our framework, reproducibility corresponds to generating responses with the same LLM and parameters for the same clinical case but across different, pre-specified diagnostic reasoning prompts. The different reasoning prompts are designed to elicit different reasoning strategies (e.g., analytic, probabilistic, intuitive), similar to how different clinicians may approach the same case from different perspectives (Table 1).

Within repeatability and reproducibility, we evaluate LLM responses along two complementary dimensions: semantic and internal metrics (Figure 1). Semantic metrics capture whether the meaning of outputs remains consistent across repeated runs, a clinically relevant property that assesses meaning rather than wording differences. Internal metrics, by contrast, quantify variability in the token-level probability distributions that underlie text generation. At each step, the model samples the next token from a probability distribution conditioned on prior context. Two outputs may appear nearly identical on the surface (e.g., “The diagnosis is meningitis” and “Diagnosis is meningitis”), yet differ substantially in their underlying token-level distributions. For example, one run may assign a high probability to the diagnosis “meningitis” while another assigns roughly equal probabilities across several diagnoses (Figure 1). Such variability across runs reflects instability in the model’s internal generation process, which may undermine its reliability in clinical applications.

### 2.2 Diagnostic Reasoning Prompts

We evaluated LLM repeatability and reproducibility across five chain-of-thought (CoT) diagnostic reasoning prompts developed and validated by Savage *et al*. [3]. These prompts were designed to elicit distinct diagnostic reasoning approaches used in clinical practice, including traditional CoT reasoning, differential diagnosis CoT, intuitive reasoning CoT, analytic reasoning CoT, and Bayesian reasoning CoT (Table 1). The full prompts used in this study are provided in Supplementary Note 1.

### 2.3 Data Sources

To evaluate the repeatability and reproducibility of LLM diagnostic reasoning across diverse clinical contexts, we selected two complementary datasets: a standardized benchmark for general medical knowledge (MedQA [19]) and real-world rare disease cases from the UDN (Table 1).

#### MedQA Dataset.

MedQA is a publicly available benchmark consisting of diagnostic clinical vignettes from the USMLE. Following Savage *et al*. [3], we used the same set of 518 questions, reformulated from multiple choice to free response format, focusing on Step 2 and Step 3 cases that emphasize clinical reasoning over rote recall. These vignettes are fully specified and standardized, making them useful for controlled evaluation of medical reasoning but somewhat idealized relative to real-world practice. An example is shown in Table 2. All questions are publicly available and provided in Supplementary Data 1 of Savage *et al*. [3].

#### Undiagnosed Diseases Network (UDN) Dataset.

To complement MedQA and address concerns that public benchmarks may have been seen during LLM pre-training, we additionally analyzed 90 non-public, diagnostically challenging rare disease cases from the Vanderbilt University Medical Center (VUMC) UDN site. In contrast to the exam-style vignettes of USMLE, UDN patients often present with complex and heterogeneous phenotypes, frequently involving multi-system manifestations, non-diagnostic clinical test results, and atypical genotypes that are challenging to interpret (e.g., variants of uncertain significance, mosaic variants, or combined genetic and non-genetic contributors). Importantly, many UDN patients had already undergone extensive prior evaluations, including subspecialist assessments and advanced testing such as exome or genome sequencing, yet remained undiagnosed. They also may have symptoms or test results that are unrelated to their final diagnosis included in the case data. Each UDN case is summarized as a multi-paragraph narrative detailing the patient’s medical history and prior workup, providing a higher-fidelity test of model performance in real-world rare disease medicine. An illustrative example is shown in Table 2. Written informed consent was obtained from all patients, and the study was approved by the VUMC Institutional Review Board (IRB# 172005).

### 2.4 Models

We performed our evaluation using three LLMs selected to represent a mix of commercial and open-source systems, model sizes, and intended use cases (Table 1). **ChatGPT-4** (**OpenAI**) is a state-of-the-art commercial LLM previously shown by Savage *et al*. to emulate clinical reasoning while maintaining high diagnostic accuracy on the same set of 518 USMLE questions [3]. To this end, we selected this model to not only evaluate its repeatability and reproducibility, but also explore whether these properties are correlated with the diagnostic accuracy metrics reported in Savage *et al*. [3]. **ChatGPT-4o-mini** (**OpenAI**) is a smaller, more cost-efficient variant that allows us to assess repeatability and reproducibility under a practical, lightweight setting. **Llama 3.2-1B** (**Meta**) is an open-source, lightweight model that provides an efficient alternative to commercial systems. Its inclusion helps evaluate the feasibility of reproducibility using lightweight models in resource-limited settings.

### 2.5 Evaluation Setup

For each of the five diagnostic reasoning prompts, we evaluated 518 USMLE cases and 90 UDN cases using each of the three LLMs, with R=100 independent runs per prompt–case–model combination, totaling 912,000 generations. ChatGPT models were accessed via the Microsoft Azure OpenAI application programming interface. To ensure patient privacy, all runs involving UDN cases were performed using a secure, institutionally sanctioned Azure OpenAI instance. We set the temperature T to 0.5, where T controls the diversity of tokens sampled in the output: values closer to 0 produce more deterministic responses, while higher values increase variability. We also used a top-k of 30, restricting sampling at each position to the 30 most likely tokens. This combination reduces the chance of incoherent responses while still allowing meaningful variability across runs. We chose these parameters to balance determinism and diversity so that both repeatability and reproducibility could be meaningfully assessed. Although we fixed these parameters for evaluation, the framework itself is agnostic to parameter choice and can be applied under any setting appropriate to the user’s application. We used two-sided multivariate Kruskal-Wallis tests at the 0.05 level to assess differences in repeatability and reproducibility scores across diagnostic reasoning prompts, datasets, and LLMs.

### 2.6 Statistical Framework for Evaluating LLM Repeatability and Reproducibility

Let X denote the input prompt and 𝒱 the LLM’s output vocabulary (i.e., set of all possible output tokens). For a given LLM and prompt, we generated R independent runs using the setting outlined in Section 2.5. Let $Y_{r,i}\in 𝒱$ denote the output token generated at position $i\;\left(i=1,\ldots ,L_{r}\right)$ in run r(r=1,…,R), where $L_{r}$ is the output length for run r. The full output sequence for run r is denoted as $Y_{r,1:L_{r}}$. To reduce noise from filler words like “an”, or “the”, we removed stopwords before calculating the metrics to focus on measuring meaningful variation.

#### 2.6.1 Semantic Repeatability

Semantic repeatability measures the consistency in the meaning of the LLM’s output across repeated runs under identical conditions. Formally, let ${𝒱}^{*}$ denote the set of all finite-length token sequences over the vocabulary 𝒱. We define an embedding function $ℰ:{𝒱}^{*}\to R^{d}$ that maps an output sequence to a d-dimensional vector representation. In this study, we used MedEmbed-Large-v1 [21] as our embedding function, which was specifically trained on clinical text and therefore was well-suited for our application. While we used MedEmbed-Large-v1 for the evaluations, our framework is agnostic to the choice of embedding model and can accommodate any model appropriate for the user’s application. For each run r=1,…,R, we compute the semantic embedding vector $e_{r}=ℰ\left(Y_{r,1:L_{r}}\right)\in R^{d}$. We then define the **Semantic Repeatability Score**, ${\tilde{S}}^{\text{Rpt}}$, as the average pairwise cosine similarity between embeddings across runs, rescaled to [0, 1] for interpretability. Larger values of ${\tilde{S}}^{\text{Rpt}}$ indicate greater semantic repeatability.

**Semantic Repeatability** (**Rpt**) **Score** (Larger = More Repeatable)

$${\overset{‾}{S}}^{\text{Rpt}}=\frac{2}{R(R-1)}\sum_{1\leq r<s\leq R}\text{cos}\left(e_{r},e_{s}\right),\;-1\leq {\overset{‾}{S}}^{\text{Rpt}}\leq 1,\;{\tilde{S}}^{\text{Rpt}}=\frac{{\overset{‾}{S}}^{\text{Rpt}}+1}{2},\;0\leq {\tilde{S}}^{\text{Rpt}}\leq 1.$$

#### 2.6.2 Internal Repeatability

Internal repeatability measures the variability in the LLM’s token-level probability distributions across repeated runs under identical conditions. In other words, it reflects the *stability of the model’s internal token*-*level generation behavior*. Lower internal repeatability indicates greater variability in the LLM’s token-level probability distributions, reflecting less stability in its output. To formalize this, let $z_{r,i}=\left(z_{r,i}\left(v\right):v\in 𝒱\right)\in R^{\left|𝒱\right|}$ denote the random vector of logits over the vocabulary 𝒱 at position i in run r. Each component $z_{r,i}(v)$ corresponds to the logit assigned to token v∈𝒱. At the first position (i=1), the logits are generated conditional only on the input prompt X, i.e., $z_{r,1}=f_{1}(X)$. For subsequent positions $\left(i=2,\ldots ,L_{r}\right)$, the logits are generated conditional on both the prompt and the sequence of previously generated tokens, i.e., $z_{r,i}=f_{i}\left(X,Y_{r,1:i-1}\right)$. These logits are transformed into a probability distribution via the temperature-scaled softmax function,

$$\pi_{r,i}(v)=\frac{\text{exp}\left\{z_{r,i}(v)/T\right\}}{\sum_{v^{\prime }\in 𝒱}\text{exp}\left\{z_{r,i}\left(v^{\prime }\right)/T\right\}},\;v\in 𝒱,$$

where T denotes the temperature ranging between 0 and 1. To focus on the subset of tokens most likely to be sampled and reduce the influence of low-probability tokens in the tails, we truncate this distribution to the top-k most probable tokens at each position, where k is a user-specified parameter and $K_{r,i}\subset 𝒱$ is the set of top-k tokens at position i in run r,

$${\tilde{\pi }}_{r,i}\left(v\right)=\{\begin{array}{ll} \pi_{r,i}\left(v\right)/\sum_{u\in K_{r,i}}\pi_{r,i}\left(u\right), & v\in K_{r,i}\\ 0, & v\notin K_{r,i} \end{array}.$$

To quantify token-level variability at position i, we compute the entropy of the LLM’s output distribution ${\tilde{\pi }}_{r,i}(\cdot )$:

$$H_{r,i}=-\sum_{v\in K_{r,i}}{\tilde{\pi }}_{r,i}\left(v\right)\;\text{log}\;{\tilde{\pi }}_{r,i}\left(v\right),$$

and average over positions in run r to obtain

$$H_{r}=\frac{1}{L_{r}}\sum_{i=1}^{L_{r}}H_{r,i}.$$

Finally, we define the **Internal Repeatability Score** ${\tilde{H}}^{\text{Rpt}}$ as the average entropy across runs, rescaled to [0, 1] for interpretability. Larger values correspond to greater repeatability.

**Internal Repeatability** (**Rpt**) **Score** (Larger = More Repeatable)

$${\overset{‾}{H}}^{\text{Rpt}}=\frac{1}{R}\sum_{r=1}^{R}H_{r},\;0\leq {\overset{‾}{H}}^{\text{Rpt}}\leq {\text{log}}_{2}k,\;{\tilde{H}}^{\text{Rpt}}=1-\frac{{\overset{‾}{H}}^{\text{Rpt}}}{{\text{log}}_{2}k},\;0\leq {\tilde{H}}^{\text{Rpt}}\leq 1.$$

#### 2.6.3 Semantic Reproducibility

Semantic reproducibility measures the consistency in the *meaning* of the LLM’s output across repeated runs using different, pre-specified prompts. In our study, we use P=5 diagnostic reasoning prompts to elicit distinct clinical reasoning strategies and evaluate whether the model produces semantically consistent outputs across these different approaches (Table 1). For each prompt p=1,…,P, we generate R independent runs and compute the semantic embeddings for each run using the embedding function ℰ described in Section 2.6.1. Let $e_{r}^{\left(p\right)}=ℰ\left(Y_{r,1:L_{r}}^{\left(p\right)}\right)$ denote the embeddings of run r under prompt p. We define the average embedding for each prompt as follows:

$${\overset{‾}{e}}^{\left(p\right)}=\frac{1}{R}\sum_{r=1}^{R}e_{r}^{\left(p\right)},\;p=1,\ldots ,P.$$

We define the **Semantic Reproducibility Score** ${\tilde{S}}^{\text{Rpd}}$ as the average pairwise cosine similarity between the prompt-specific mean embeddings, rescaled to [0, 1] for interpretability. Larger values indicate greater reproducibility of semantic meaning in the LLM’s output across different diagnostic reasoning prompts.

**Semantic Reproducibility** (**Rpd**) **Score** (Larger = More Reproducible)

$${\overset{‾}{S}}^{\text{Rpd}}=\frac{2}{P(P-1)}\sum_{1\leq p<q\leq P}\text{cos}\left({\overset{‾}{e}}^{\left(p\right)},{\overset{‾}{e}}^{\left(q\right)}\right),\;-1\leq {\overset{‾}{S}}^{\text{Rpd}}\leq 1,\;{\tilde{S}}^{\text{Rpd}}=\frac{{\overset{‾}{S}}^{\text{Rpd}}+1}{2},\;0\leq {\tilde{S}}^{\text{Rpd}}\leq 1.$$

#### 2.6.4 Internal Reproducibility

Internal reproducibility measures the variability in the LLM’s token-level probability distributions across repeated runs with different, pre-specified prompts. In other words, it evaluates *how robust the model’s token*-*level generating process is to variation in diagnostic reasoning prompts*. For each prompt p=1,…,P, we generate R independent runs and compute the average entropy. Let $H_{r}^{\left(p\right)}$ denote the mean entropy of the top-k output tokens in run r under prompt p. We define the average entropy for each prompt as:

$${\overset{‾}{H}}^{\left(p\right)}≔\frac{1}{R}\sum_{r=1}^{R}H_{r}^{\left(p\right)},\;p=1,\ldots ,P.$$

We then compute the **Internal Reproducibility Score ${\tilde{H}}^{\text{Rpd}}$** as the average absolute difference in mean entropy across all prompt pairs, rescaled to [0, 1] for interpretability. Larger values indicate higher reproducibility.

**Internal Reproducibility** (**Rpd**) **Score** (Larger = More Reproducible)

$${\overset{‾}{H}}^{\text{Rpd}}=\frac{2}{P(P-1)}\sum_{1\leq p<q\leq P}\left|{\overset{‾}{H}}^{\left(p\right)}-{\overset{‾}{H}}^{\left(q\right)}\right|,\;0\leq {\overset{‾}{H}}^{\text{Rpd}}\leq {\text{log}}_{2}k,\;{\tilde{H}}^{\text{Rpd}}=1-\frac{{\overset{‾}{H}}^{\text{Rpd}}}{{\text{log}}_{2}k},\;0\leq {\tilde{H}}^{\text{Rpd}}\leq 1.$$

## 3 Results

### 3.1 Repeatability and Reproducibility

Overall, both semantic and internal repeatability varied across diagnostic reasoning prompts, models, and datasets (Figure 2). For ChatGPT-4, the Bayesian CoT prompt resulted in higher semantic repeatability on both the USMLE and UDN cases, indicating that probabilistic reasoning may help the model generate more consistent clinical interpretations across runs. In contrast, internal repeatability scores were relatively consistent across models, prompts, and datasets. An exception was ChatGPT-4o-mini, where the Traditional CoT and Bayesian CoT prompts resulted in substantially lower internal repeatability. Compared to USMLE cases, repeatability scores for UDN cases showed less variation across prompts, suggesting that the complexity of real-world cases may constrain the LLM’s output space and reduce prompt sensitivity. This trend was consistent across all LLMs.

Similarly, reproducibility scores also varied less for UDN than for USMLE cases, a trend observed for all three LLMs (Figure 2). On the USMLE cases, ChatGPT-4o-mini generally achieved the highest internal reproducibility, indicating consistent token-level generation behavior across different prompts. In contrast, Llama 3.2-1B achieved the highest semantic reproducibility, suggesting greater robustness in the overall meaning of its outputs across different prompts. For the UDN cases, internal reproducibility was relatively uniform across all three models. However, Llama 3.2-1B outperformed the others in semantic reproducibility, indicating that its responses were more stable semantically in real-world cases. These findings suggest that lightweight models like Llama 3.2-1B may exhibit stronger semantic robustness to prompt variation in complex clinical cases, possibly because their reduced capacity limits overfitting to prompt-specific patterns.

### 3.2 Relationship among LLM repeatability, reproducibility, and diagnostic accuracy

We assessed whether repeatability and reproducibility were correlated with diagnostic accuracy using published ground-truth labels for ChatGPT-4 on USMLE cases from Savage *et al*. [3]. Briefly, these labels were created by physicians who manually reviewed ChatGPT-4’s outputs to determine diagnostic accuracy (see Supplement 1 in Savage *et al*. [3]). As shown in Figure 3, across four of five prompts, traditional CoT, differential diagnosis CoT, analytic CoT, and Bayesian CoT, there was no statistically significant difference in repeatability scores between correctly and incorrectly diagnosed cases. For the intuitive CoT prompt, however, higher internal repeatability scores were positively associated with diagnostic accuracy (p-value <0.001). This suggests that for certain reasoning paradigms, greater token-level consistency may correlate with improved accuracy. Figure 3 shows reproducibility scores for the same cases. There was no statistically significant association between reproducibility and diagnostic accuracy, suggesting that accurate diagnoses do not necessarily correlate with consistent outputs across different prompts.

## 4 Discussion

We developed a generalizable framework for assessing the repeatability and reproducibility of LLMs and applied it to a use case on diagnostic reasoning. A key finding was that repeatability and reproducibility scores were less variable for UDN cases than USMLE questions, suggesting that the ambiguity and complexity of real-world patient presentations may constrain the model’s output space and make its reasoning less sensitive to how the prompt is phrased. This may be because LLMs have not seen the UDN cases during pre-training (as these data are not publicly available), and without familiar or memorized patterns to draw from, the model falls back on more generic reasoning strategies that yielded more stable outputs across prompts. This aligns with recent evidence that LLMs rely heavily on pattern matching rather than genuine reasoning in medicine [22]. In addition, the longer and more detailed structure of UDN cases, which is common in real-world clinical documentation, may also contribute to this finding. In addition, we observed that prompts invoking probabilistic (Bayesian) reasoning yielded higher semantic repeatability for ChatGPT-4, underscoring that repeatability is influenced not only by the model itself but also by how reasoning is elicited. These findings illustrate that LLM reliability is not a fixed property but depends on the interplay among the model, prompt, and dataset used for evaluation. As such, performance results should not be over-generalized across settings, and reliability metrics, including repeatability and reproducibility, should be established for each intended application.

Another key finding was that, in general, repeatability and reproducibility did not correlate with diagnostic accuracy. This highlights a critical distinction: producing a correct response is not equivalent to producing that response consistently. An LLM that generates variable outputs, even if accurate on average, may undermine clinician trust, disrupt decision-making, and limit the model’s utility in patient care. Therefore, evaluating repeatability and reproducibility alongside accuracy is necessary to determine whether an LLM is ready for clinical use, ensuring that outputs are not only correct but also reliable in practice. Notably, studies show that LLMs can underperform in forecasting tasks compared to traditional machine learning [23] and may rely on pattern recognition rather than medical reasoning [22], underscoring that there are robustness gaps in LLM performance and systematic evaluation requires metrics beyond accuracy alone.

This work is distinct from and complementary to prior efforts on quantifying and improving LLM consistency in the field of natural language processing. Traditional reference-based semantic metrics such as BLEU, ROUGE, and BERTScore evaluate similarity to a gold-standard output, whereas our framework quantifies a model’s self-consistency and stability across repeated runs as a complementary measure of reliability [24–26]. Raj *et al*. [27] proposed semantic consistency metrics and demonstrated that consistency and accuracy are independent properties, an observation that aligns with our findings in the clinical domain. Similarly, Cui *et al*. [28] introduced a divide-and-conquer approach to improve sentence-level consistency, showing that such metrics can serve as both evaluative tools and levers for model refinement. Wang *et al*. [29] proposed MONITOR, a framework for assessing factual reliability under prompt variability, further underscoring the limitations of accuracy as the sole evaluation metric. More recently, Raj *et al*. [30] introduced Chain of Guidance, a guided prompting strategy that substantially improves semantic consistency in question-answering tasks. Complementary to these works in the general domain, our study focuses on diagnostic reasoning in medicine and evaluates FDA-defined properties of repeatability and reproducibility, providing a step towards regulatory-aligned evaluation of clinical LLMs. This has direct implications for building clinician trust, assessing diagnostic reliability, and ensuring patient safety.

This work has several limitations that we highlight for future investigation. First, due to the substantial computational burden of generating repeated outputs across prompts, models, and clinical cases, our evaluation prioritized breadth across model types and clinical scenarios rather than exhaustive coverage of all possible configurations. While we selected diverse models, reasoning paradigms, and datasets to reflect real-world clinical variation, additional clinical contexts should be considered. A second potential limitation of this work is that our semantic consistency evaluations used a specific embedding model to quantify output meaning. While results may vary across embedding spaces, all evaluations in this study used the same embedding model to ensure fair and consistent comparisons. Still, future studies should explore the sensitivity of semantic reproducibility scores to alternative embeddings.

Future extensions of this work include incorporating methods for assessing clinical validity. Our framework could be adapted to integrate techniques from hallucination detection and factuality checking, such as sampling-based consistency or retrieval-grounded evaluation [18, 31–33], to determine whether consistent outputs are evidence-based. Such approaches would allow consistency metrics to serve not only as evaluative tools, but also as safeguards for ensuring that repeated outputs remain factually correct and clinically actionable. As LLMs are increasingly trained with advanced reinforcement learning methods, self-verification, and outcome-based reasoning optimization [34–36], evaluating how these techniques impact the reliability of model output will be critical for their responsible use in medicine. Taken together, this line of work can help establish standards for determining when LLMs achieve the accuracy, repeatability, and reproducibility required for reliable use in clinical applications and biomedical research, laying a foundation for rigorous evaluation to guide clinical integration and responsible innovation of AI in medicine.

## Supplementary Material

Supplement 1

## Figures and Tables

**Fig. 2A.**
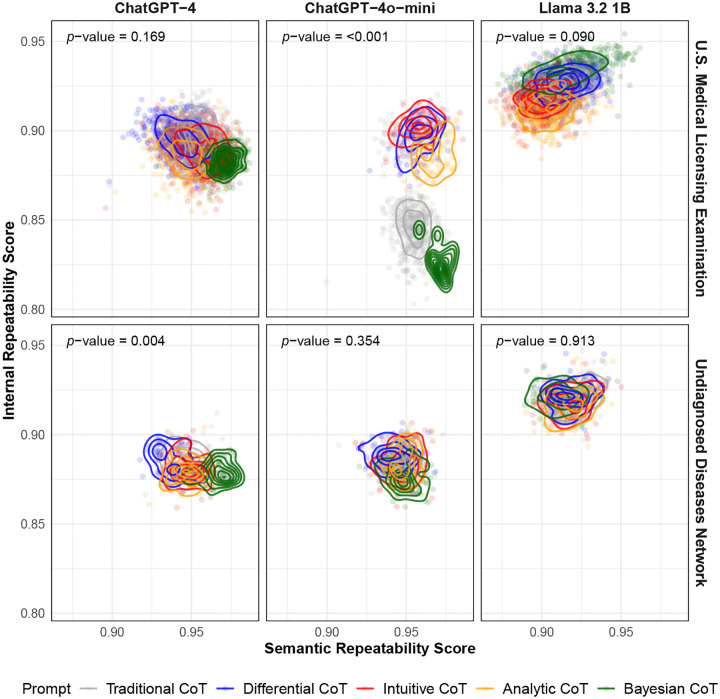
Semantic and internal repeatability scores by model, dataset, and prompt. Each point represents a single clinical case evaluated under a specific prompt, plotted by its semantic repeatability score (x-axis) and internal repeatability score (y-axis). Contour lines indicate the 2-dimensional density of case-level scores within each prompt category. Plots are faceted by model (columns) and dataset (rows). p-values are calculated based on a two-sided multivariate Kruskal–Wallis test at the 0.05 level. Larger is better (more repeatable) for both axes. *CoT* = chain-of-thought; *UDN* = Undiagnosed Diseases Network.

**Fig. 2B.**
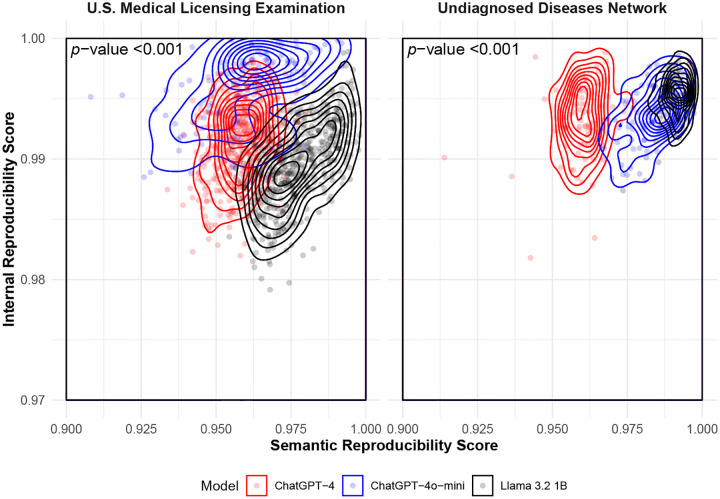
Semantic and internal reproducibility scores by model and dataset. Each point represents a single clinical case evaluated by a given model, with semantic reproducibility (x-axis) and internal reproducibility (y-axis) computed across the five prompts in Table 1. Contour lines represent 2-dimensional kernel density estimates of case-level reproducibility scores within each model. Left panel = USMLE dataset. Right panel = UDN dataset. p-values are calculated based on a two-sided multivariate Kruskal–Wallis test at the 0.05 level. Larger is better (more reproducible) for both axes. *UDN* = Undiagnosed Diseases Network.

**Fig. 3A.**
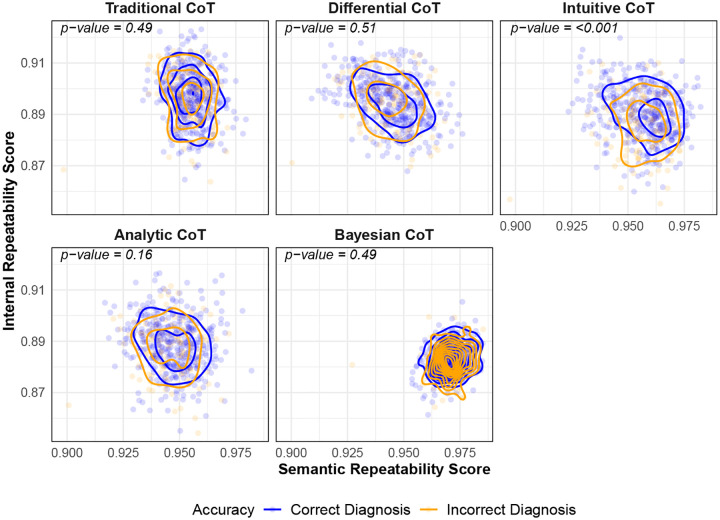
Semantic and internal repeatability scores for ChatGPT-4 on the MedQA (U.S. Medical Licensing Examination) dataset stratified by prompt. Each point represents a single clinical case evaluated by ChatGPT-4, with semantic repeatability score on the x-axis and internal repeatability score on the y-axis. Contour lines indicate the 2-dimensional density of case-level scores for cases that were correctly diagnosed by ChatGPT-4 (blue) and incorrectly diagnosed by ChatGPT-4 (orange). p-values are calculated based on a two-sided multivariate Kruskal-Wallis test at the 0.05 level. Larger is better (more repeatable) for both axes. *CoT* = chain-of-thought.

**Fig. 3B.**
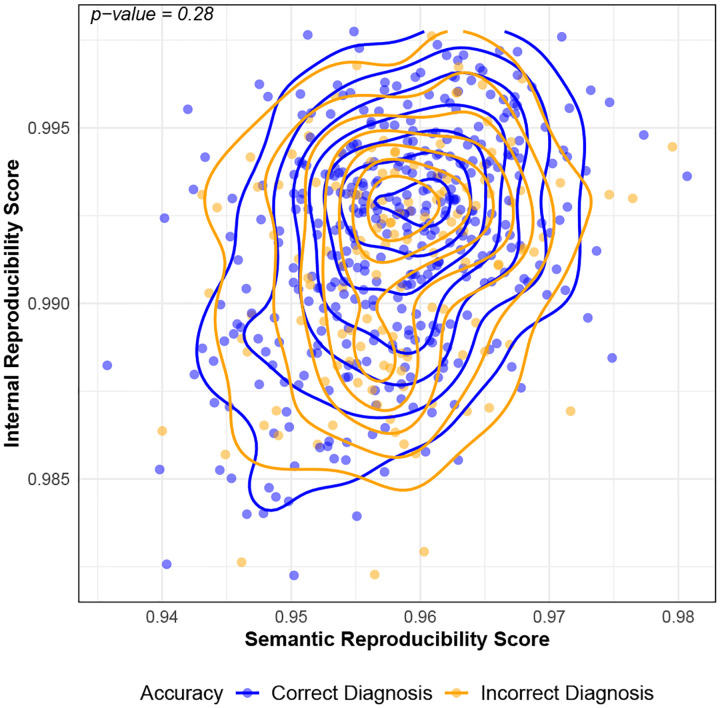
Semantic and internal reproducibility scores for ChatGPT-4 on the MedQA (U.S. Medical Licensing Examination) dataset. Each point represents a single clinical case evaluated by ChatGPT-4, with semantic reproducibility score on the x-axis and internal reproducibility score on the y-axis computed across the five prompts in Table 1. Contour lines indicate the 2-dimensional density of case-level scores for cases that were correctly diagnosed by ChatGPT-4 (blue) and incorrectly diagnosed by ChatGPT-4 (orange). p-values are calculated based on a two-sided multivariate Kruskal-Wallis test at the 0.05 level. Larger is better (more reproducible) for both axes.

**Table 1:** Overview of evaluation framework. CoT = chain of thought; USMLE = U.S. Medical Licensing Examination; NIH = National Institutes of Health.

Prompt Name	Prompt from Savage *et al*.[3]	Reasoning Paradigm
Traditional CoT	Provide a step-by-step deduction that identifies the correct response.	General logical reasoning aimed at identifying the correct answer.
Differential Diagnosis CoT	Use step-by-step deduction to create a differential diagnosis and then determine the correct response.	Clinical reasoning incorporating differential diagnosis before narrowing it down to the final diagnosis.
Intuitive Reasoning CoT	Use symptoms, signs, and lab associations to step-by-step deduce the correct response.	Heuristic reasoning grounded in clinical pattern recognition and associations.
Analytic Reasoning CoT	Use analytic reasoning to deduce the pathophysiology and step-by-step identify the diagnosis.	Mechanistic reasoning focused on underlying biological or physiological processes.
Bayesian Reasoning CoT	Use Bayesian inference to create a prior, update with new information, and determine the diagnosis.	Probabilistic reasoning involving dynamic updating of diagnostic likelihoods based on new evidence.
Data	MedQA[19] USMLE (n=518)	Rare Disease Cases (n=90)
Source	Public benchmark	NIH Undiagnosed Diseases Network
Content	Standardized exam-style vignettes	Real-world patient cases
Completeness	Fully specified questions	Often incomplete or open-ended
Clinical Context	General medical knowledge	Atypical, complex, and heterogeneous cases
Realism	Synthetic and often idealized	High-fidelity, real clinical data
LLM	Type	Rationale
ChatGPT-4	Commercial	Shown to emulate clinical reasoning without loss in diagnostic accuracy [3]
ChatGPT-4o-mini	Commercial	Lightweight and cost efficient
Llama 3.2-1B	Open Source	Lightweight and efficient

**Table 2:** Example of USMLE and UDN clinical cases. USMLE = U.S. Medical Licensing Examination; UDN = Undiagnosed Diseases Network.

**USMLE Case:** A 55-year-old man comes to the physician because of a 6-week history of tingling pain in the sole of his right foot when he raises it above chest level during exercises. He reports that he started exercising regularly 2 months ago and that his right calf cramps when he uses the incline feature on the treadmill, forcing him to take frequent breaks. The pain completely disappears after resting for a few minutes. He has an 8-year history of type 2 diabetes mellitus. He has smoked two packs of cigarettes daily for 34 years. His only medication is metformin. His pulse is 82/min, and blood pressure is 170/92 mm Hg. Straight leg raise test elicits pallor and tingling pain in the right foot. There is no pain in the back. His muscle strength is normal. Femoral pulses are palpable; right pedal pulses are absent.
**UDN Case:**
**“One-liner”:** 6-year-old male with short stature, developmental delay, dysmorphic facial features, cryptorchidism, and pulmonary valve stenosis.
**Category of Primary Condition:** Neurology
**Narrative Summary:** At birth, noted to have generalized hypotonia, poor suck, and distinct facial features. Required NG feeding for the first two weeks of life. Developmental delays were evident from infancy. Rolled at 8 months, sat independently at 15 months, walked at 3 years. Now 6 years old, speaks only a few single words. Formal developmental assessment confirmed global developmental delay. Behavioral profile includes repetitive hand movements, mild incoordination, and attention difficulties.
**Medical history notable for:**
Pulmonary valve stenosis (stable)
Bilateral cryptorchidism (surgically repaired)
Recurrent otitis media (tubes ×2)
Severe constipation (daily laxatives)
**Percentiles:**
Height: <3rd percentile
Weight: 5th percentile
Head circumference: 10th percentile
**Facial Features and Measurements:**
Hypertelorism (intercanthal distance: 3.6 cm, 95th percentile)
Down-slanting palpebral fissures
Ptosis
Low-set, posteriorly rotated ears (Right: 4.2 cm, Left: 4.3 cm; both ¡3rd percentile)
Broad/webbed neck
Shield chest with widely spaced nipples
High anterior hairline
**Family History:**
Father: Short stature and had a heart murmur in childhood, never fully evaluated
Paternal grandmother: Described as having a similar facial appearance
Mother: Healthy
**Prior Genetic Testing:**
Chromosomal Microarray: Normal
Fragile X testing: Negative
Whole Exome Sequencing (trio): VUS in PTPN11, currently under review
Karyotype: 46,XY
**Known Prior Evaluations:**
Brain MRI (2023): Normal
Echocardiogram: Mild-to-moderate pulmonary valve stenosis, stable
Audiology: Mild conductive hearing loss (likely secondary to otitis media)

## Data Availability

The MedQA (U.S. Medical Licensing Examination) cases used in this study are publicly available in Savage *et al*. [3]’s Supplementary Data 1. The Undiagnosed Diseases Network data used in this study contain sensitive patient information. De-identified patient data, including phenotypic and genomic data, are deposited in the database of Genotypes and Phenotypes (dbGaP) maintained by the National Institutes of Health. To explore data available in the latest release, visit the UDN study page in dbGaP. Individuals interested in accessing UDN data through dbGaP should submit a data access request. Detailed instructions for this process can be found on the NIH Scientific Data Sharing website: How to Request and Access Datasets from dbGaP.

## Code availability

Code used in this study are publicly available at https://github.com/cathyshyr/repeatability_and_reproducibility_of_LLMs.
